# More than half of hypoxemia cases occurred during the recovery period after completion of esophagogastroduodenoscopy with planned moderate sedation

**DOI:** 10.1038/s41598-020-61120-0

**Published:** 2020-03-09

**Authors:** Yukihiro Shirota, Yoshimi Hirase, Tsuyoshi Suda, Masaki Miyazawa, Yuji Hodo, Tokio Wakabayashi

**Affiliations:** 1 Department of Gastroenterology, Saiseikai Kanazawa Hospital, Kanazawa, Japan; 2 Department of Nursing, Saiseikai Kanazawa Hospital, Kanazawa, Japan

**Keywords:** Oesophagogastroscopy, Adverse effects

## Abstract

Guidelines advise precautionary measures for possible adverse events that may occur due to sedation during endoscopic procedures. To avoid complications, intraprocedural and postprocedural monitoring during recovery is considered important. However, since not many studies have reported on hypoxemia during the recovery period, findings for specific monitoring methods are insufficient. The aim of this retrospective study was to determine the incidence of hypoxemia during the recovery period using continuous central-monitoring by pulse oximetry and to characterize the hypoxemia cases. Among the 4065 consecutive esophagogastroduodenoscopy (EGD) procedures under planned moderate sedation, 84 (2.1%) procedures developed unexpected hypoxemia (SpO_2_ ≤ 90%). Hypoxemia was observed during the procedure, at the end of the procedure, and during the recovery period in 21, 17, and 46 (1.1%) procedures, respectively. More than half of the hypoxemia cases occurred during the recovery period. Many hypoxemia cases were characterized by neither serious co-morbid illness nor low body mass index which have been reported as risk factors of hypoxemia. The lack of risk factors is no guarantee that hypoxemia will not occur. Therefore, continuous monitoring by pulse oximetry is more important during the recovery period and is recommended in all EGD procedures under planned moderate sedation.

## Introduction

The American Society for Gastrointestinal Endoscopy (ASGE) and the European Society of Gastrointestinal Endoscopy (ESGE) established guidelines in the early 2000’s for the appropriate use of sedation during endoscopic procedures^1,2^. Sedatives had also been used for endoscopic procedures in Japan, but the Japan Gastroenterological Endoscopy Society (JGES) and the Japanese Society of Anesthesiologists (JSA) jointly issued their “Guidelines for sedation in gastroenterological endoscopy”^3^ in 2013. The guidelines state that, “Sedation in endoscopic procedures has the benefit of eliminating anxiety and discomfort in patients and improving patient acceptance of and satisfaction with endoscopic procedures”, and they also note that, “From the viewpoint of endoscopists, sedation is useful for improving the completion rate, quality of endoscopic examination and treatment outcomes of therapeutic endoscopy.” Furthermore, the adequate target sedation level in routine endoscopic examination such as esophagogastroduodenoscopy (EGD) without treatment is set to moderate rather than deep as defined by the American Society of Anesthesiologists (ASA)^4^, and it recommends that, “In endoscopy under sedation, it is important to inspect patients and appropriately monitor their level of consciousness and cardiorespiratory dynamics. Patient monitoring should be continued until complete recovery even after the completion of endoscopic procedures.”

However, due to the lack of actual clinical data, specific details on the methods for conducting postprocedural recovery monitoring have not been examined sufficiently, even in the recently updated ASGE guidelines in 2014 and 2018^5,6^. Furthermore, studies of the most commonly performed^7^ EGD procedures are virtually non-existent. Regarding the frequency of adverse events, a survey by JGES^7^ based on a total of 17,087,111 endoscopic procedures found that 219 adverse events were related to sedatives and analgesics. However, as the total number of endoscopic procedures included many that were conducted without using sedatives or analgesics, the frequency of adverse events among procedures conducted using sedatives is unknown. The aim of this study was to determine the incidence of adverse events during the recovery period and to assess the effectiveness of continuous monitoring by conducting a retrospective analysis of the data.

## Results

Of the 7332 EGD procedures conducted between April 1, 2015 and December 31, 2016, there were 4065 consecutive outpatient EGD procedures conducted under sedation in 2890 unique patients (Table 1). The age range of the patients for the 4065 EGD procedures was between 15 and 92 years (median 57 years), and the ratio of men to women was 2483 to 1582. As for the reason for seeking consultation, 1688 EGD procedures were conducted due to some kind of symptom or disorder, and 2377 EGD procedures were conducted as part of the medical check-ups systems established in Japan with a high incidence of gastric cancer as both population-based and opportunistic screening. In terms of the sedative used, diazepam and midazolam were used in 4049 (99.6%) and 16 (0.4%) EGD procedures, respectively, and pentazocine was added in eight procedures (0.2%) (Table 2).

**Table 1:** Total number of evaluable cases and number of procedures that developed hypoxemia.

Total EGD procedures	7332
Consecutive outpatient EGD procedures with sedation	4065
Unique patients	2890
Procedures that developed unexpected hypoxemia, SpO_2_ ≤ 90%	84 (2.1%)
Unique patients	72 (2.5%)
with a history of using the same type and dose of sedatives	41
and with a history of no hypoxemia	29

**Table 2:** Patient background characteristics and drugs used among the 4065 consecutive outpatient EGD procedures with sedation.

Age (years)	15–92 (median 57)
Sex (men:women)	2483:1582
Reasons for consultation (procedures)	
Digestive disease (outpatient visits)	1688
Medical check-ups	2377
Sedatives/analgesics used (procedures)	
Diazepam	4049 (99.6%)
Midazolam	16 (0.4%)
Supplemental pentazocine	8 (0.2%)
Combined with diazepam	5
Combined with midazolam	3

Of these procedures, there were 84 (2.1%) procedures and 72 unique patients (2.5%) who developed unexpected hypoxemia (SpO_2_ of ≤90%) between the intraprocedural and recovery periods (Table 1). The age of the patients at the time of these 84 hypoxemia cases ranged between 38 and 88 years (median 71.5 years). The ratio of men to women for the procedures was 1:1. Eastern Cooperative Oncology Group Performance Status (ECOG-PS) Grades were 0/1/2/3/4 in 33/40/10/1/0 patients, respectively. The mean height was 157.9 cm (135.6 to 186.7 cm), mean body weight was 64.1 kg (32.0 to 97.7 kg), and mean body mass index (BMI) was 25.6 (14.1 to 35.9) kgm^−2^ (data were confirmed for 74 of 84 hypoxemia cases). The number of patients with co-morbid illnesses that could affect cardiorespiratory dynamics or the metabolism of the sedatives were as follows: 11 respiratory disease patients (chronic respiratory failure, chronic obstructive pulmonary disease, asthma, interstitial pneumonia, and pulmonary emphysema); five cardiovascular disease patients (ischemic heart disease); five kidney disease patients (renal dysfunction, chronic kidney disease, and chronic kidney failure on maintenance hemodialysis); 24 liver disease patients (viral chronic hepatitis, autoimmune hepatitis, non-alcoholic fatty liver disease, alcoholic liver disease, compensated and uncompensated liver cirrhosis); and 48 patients that did not have any of these illnesses of 84 hypoxemia cases. The American Society of Anesthesiologists Physical Status (ASA-PS) Classes were I/II/III/IV/V in 28/43/13/0/0 procedures, respectively (Table 3). The sedative used in 82 of 84 hypoxemia cases was diazepam [mean dose 6.30 mg, men 6.95 (5 to 7.5) mg, women 5.93 (2.5 to 7.5) mg], and two procedures used midazolam [women only, mean dose 5.00 (4.0 to 6.0) mg]. None of the cases received analgesics such as pentazocine. The mean duration of the EGD procedure was 7 minutes and 41 seconds (5 to 35 min).

**Table 3:** Patient background characteristics and drugs used in the 84 procedures that developed hypoxemia and EGD procedure time.

Age (years)	38–88 (median 71.5)
Range 30–39/40–49/50–59/60–69/70–79/80–89	1/4/14/20/35/10
Sex (men:women)	1:1
ECOG-PS Grade 0/1/2/3/4	33/40/10/1/0
Height (cm)*	Mean 157.9 (135.6–186.7)
Weight (kg)*	Mean 64.1 (32.0–97.7)
BMI (kgm^−2^)*	Mean 25.6 (14.1–35.9)
BMI>30	11
BMI<18.5	3
Underlying medical conditions	
Respiratory disease	11 (13.1%)
Cardiovascular disease	5 (6.0%)
Liver disease	24 (28.6%)
None of the above diseases	48 (57.1%)
ASA-PS Class I/II/III/IV/V	28/43/13/0/0
Type and dose of sedative used	
Diazepam	82
Mean dose (mg)	6.30
Men	6.95 (5–7.5)
Women	5.93 (2.5–7.5)
Midazolam	2 (only women)
Mean dose (mg)	5.00 (4.0–6.0)
Supplemental pentazocine	0
EGD procedure time	Mean 7 min 41 sec (5 to 35 min)

*Data confirmed in 74 procedures.

**Figure 1:**
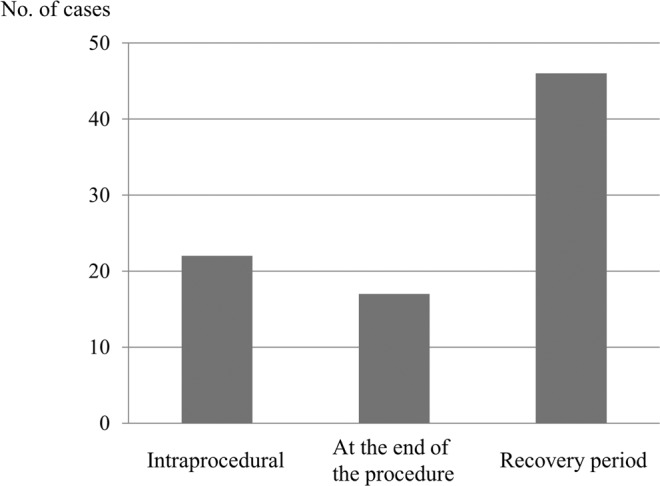
Timing of hypoxemia onset.

**Figure 2:**
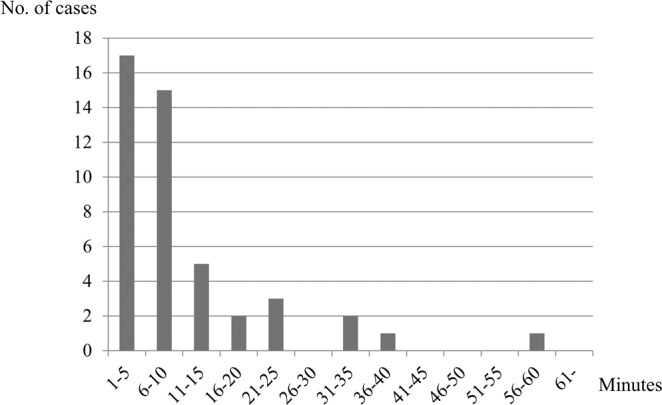
Time from the end of the procedure to the onset of hypoxemia during the recovery period.

Hypoxemia developed intraprocedurally, at the end of the procedure, and during the recovery period in 21, 17, and 46 procedures, respectively (Fig. 1). More than half of the cases occurred during the recovery period, accounting for 1.1% of the 4065 EGD procedures that were performed under sedation. Among the 46 procedures that developed hypoxemia during the recovery period, the mean time in which hypoxemia occurred was 12 minutes and 53 seconds after the end of the procedures. There were 39 cases (84.8%) (17, 15, 5, and 2 cases occurred between 1 to 5 minutes, 6 to 10 minutes, 11 to 15 minutes, and 16 to 20 minutes, respectively), and over 80% of the cases experienced hypoxemia within 20 minutes, whereas seven cases (15.2%) of hypoxemia occurred between 21 and 60 minutes (Fig. 2). The mean recovery period was 1 hour, 9 minutes, and 45 seconds (minimum 40 minutes and maximum 2 hours and 20 minutes) (Table 4). In Japan, an annual examination is not uncommon. When the scope of examination of the EGD records at our institution was expanded to July 31, 2017, the same sedative with the same dose was administered in 41 of the 84 procedures that developed hypoxemia. Of these procedures, the records showed that hypoxemia did not develop in 29 unique patients (Table 1).

**Table 4:** Method used to detect the 84 hypoxemia (procedures), follow-up response, outcome, and duration of the recovery period.

Method of detection (procedures)	
Pulse oximetry with an audible alarm	84
Visual observation of apnea by healthcare personnel	0
Response (procedures)	
Oxygen administration	84
Unplanned tracheal intubation	0
Advanced life support	0
Administration of flumazenil	0
Outcome (procedures)	
Recovery	84
Hospitalization	0
Subsequent complications	0
Death	0
Duration of recovery period	Mean 1 h 9 min 45 sec (40 min to 2 h 20 min)

Oxygen was administered in all hypoxemia cases. Of the 46 hypoxemia cases that occurred during the recovery period, none complained of coughing up sputum and needed sputum suction during recovery. There were no serious adverse events that required unplanned tracheal intubation or advanced life support (cardiopulmonary resuscitation in cardiac arrest and related conditions). There were also no cases that required hospitalization or resulted in subsequent complications. There were also no cases that required flumazenil, an antagonist agent. In all hypoxemia cases, hypoxemia was detected intraprocedurally, at the end of the procedure, or during the recovery period by an audible alarm that was triggered due to a drop in SpO_2_, and not through visual observation of apnea by healthcare personnel (Table 4). There were no cases of tachycardia or bradycardia that required treatment intervention.

## Discussion

The effectiveness of sedation during endoscopic procedures is noted and widely used in the guidelines in the USA, Europe, and Japan. However, there have been reports of adverse events related to sedation, and therefore, countermeasures are needed. A survey of adverse events in Japan^7^ found that, of the 472 adverse events related to preparations such as sedation, pharyngeal anesthesia, and intestinal irrigation, 219 (46.4%) were related to sedatives and analgesics. Of the 175 events recorded in the case records, the most frequently mentioned events were respiratory depression and arrest (99 events), followed by 22 adverse events of hypoxemia. Four of these adverse events resulted in death. Based on a total of 17,087,111 endoscopic procedures that were examined in the above study, the frequency of preparation-associated adverse events was 0.0028%. However, since the total number of endoscopic procedures included many that were conducted without using sedatives or analgesics, the frequency of adverse events among procedures conducted using sedatives is unknown. In the present study, of the 4065 EGD procedures that were conducted under planned moderate sedation, 84 developed hypoxemia in the course of the entire process from the intraprocedural period to the recovery period, accounting for a high proportion, at 2.1%.

Three-quarters of the 84 procedures that developed hypoxemia occurred either at the end of the procedure or during the recovery period. Endoscopists might be surprised at this observation. Coughing and aspiration upon insertion of the endoscope during a procedure may at times induce hypoxemia; however, these events are temporary. In addition, the stimulation caused by insertion of the endoscope promotes arousal and makes it less likely to cause respiratory depression especially in moderate sedation. On the other hand, removal of the endoscope may provide relief from stimulation, deepening the sedation and facilitating the development of hypoxemia after the end of the procedure. Being aware of the timing of onset of hypoxemia is important in managing respiratory depression during EGD procedures under moderate sedation. During the procedure, experienced healthcare personnel such as physicians and nurses are by the patient’s side, and they can monitor the patients during non-complicated procedures such as EGD. However, during the recovery period, if monitoring equipment is not used, patient observation may be intermittent even if designated healthcare personnel are allocated to monitor the patient in recovery. Hypoxemia due to respiratory depression may not be detected between observations before critical situations such as respiratory arrest were to occur.

The sedative used in most procedures in the present study was diazepam. Many randomized, controlled trials (RCTs)^8–12^ have demonstrated that there is no difference in side effects such as cardiorespiratory depression between diazepam and midazolam when used during EGD procedures. When the recovery times of diazepam and midazolam were compared, midazolam was found to have a faster recovery^10^. However, since another study^11^ found that diazepam has a faster recovery if recovery occurs within 1 hour, there is not yet a unified view. It is a mistake to assume that the frequency and trends of hypoxemia cases during the recovery period in this study can be specifically attributed to diazepam. However, one can say that it is a common phenomenon associated with benzodiazepines, including midazolam, and requires preventive measures against adverse effects.

There are differences in recovery monitoring methods among the USA, European, and Japanese guidelines. The JGES guidelines in 2015^3^ and the ASGE guidelines in 2003^1^ do not specify the details of how to conduct the monitoring. The ESGE guidelines in 2008^2^ specify that, “Close monitoring of the patient by qualified personnel should be continued, irrespective of the substance used, and using a pulse oximeter if thought desirable, until the patient has completely recovered.” Even though the use is restrictive, the guidelines recommend monitoring with a pulse oximeter. But the cited literature is also part of the guidelines^13^, and so the evidence is poor. The updated version of the ASGE guidelines in 2014^5^ states that minimal monitoring requirements include electronic assessment of pulse oximetry combined with visual monitoring of the patient’s level of consciousness and discomfort. However, these guidelines state that the monitoring should be performed at regular intervals rather than continuously during procedure, during initial recovery, and just before discharge. On the other hand, the ASGE training guidelines^14^ outline in detail the monitoring method of propofol when used for deep sedation as follows: “Continuous monitoring of pulse oximetry, vital signs, respiratory function, and consciousness is appropriate and should be documented at regular intervals until patients have returned to or approached their baseline status.” A PubMed search using the keywords “endoscopy”, “sedation”, and “hypoxemia (or hypoxia) in recovery” retrieved 47 studies, and there was only one study^15^ that reported using a pulse oximeter during the recovery period. This study involved 30 patients aged 60 years or older who underwent ERCP using midazolam. The patients were monitored before, during, and for 2 hours after the procedure using pulse oximeters to examine the changes in oxygen saturation over time. According to this study, patients were most hypoxic in the first 30 minutes after the procedure; however, this was an RCT with a small sample size that was separated into two groups, with one group receiving flumazenil and the other group receiving normal saline, and it was not a study of hypoxemia cases. As noted above, monitoring methods during the recovery period differ between the different guidelines, and this is most likely due to the lack of evidence. While the data used in the present study were retrospective in nature, they are highly significant as real-world data due to the large sample size. It is important to emphasize that this study targeted non-propofol cases in which a moderate level and not a deep level of sedation was planned. Even in cases where moderate sedation is planned using non-propofol sedatives, continuous monitoring is needed during the recovery period when considering the frequency and onset of respiratory depression during the recovery period.

Regarding the prognosis of hypoxemia cases, there were no serious adverse events that required advanced life support or hospitalization by monitoring using pulse oximetry without standard periodic rounds by dedicated healthcare personnel in recovery period. According to a review^16^ of specific monitoring methods conducted by the American Gastroenterological Association (AGA) in 2008, “Measurement of oxygen saturation is supplemental to clinical observation of the patient.”, the review stated the following reasons for their recommendation: (i) measurement of oxygen saturation is relatively insensitive to the earliest signs of hypoventilation; and (ii) the inability to detect an adequate signal during hypothermia, low cardiac output, and motion (e.g. tremor). It also mentioned that the ability of oximetry to reduce the incidence of cardiopulmonary complications remains unproven. While monitoring with pulse oximetry may be a complementary requirement, as noted in the review, it is not a sufficient requirement. However, it is important to take note of the fact that a large number of cases (4065 patients) were monitored using pulse oximetry during the recovery period in the present study, and in the 84 cases that developed hypoxemia, measures were taken before serious adverse events that require care such as unplanned tracheal intubation or advanced life support occurred. In addition, the onset of hypoxemia of all 84 cases was identified by the audible alarm detecting a decline in SpO_2_. The subjects of this study were outpatients who underwent EGD with planned moderate sedation, and it was a group of patients with relatively good performance status. Even the 84 cases that developed hypoxemia were all categorized as ASA-PS Class III or lower, with the majority (84.5%) in Classes I and II. If the patient undergoing an EGD procedure with planned moderate sedation is categorized as at least ASA-PS Class II or lower, monitoring by pulse oximetry may sufficiently meet the conditions for preventing possible serious adverse events. For cases that are ASA-PS Class III or higher, careful consideration must be given towards not only the monitoring methods during the recovery period, but also the actual procedure itself and the use of sedatives.

Two studies^17,18^ examined the relationships between ASA-PS classification and adverse events related to endoscopic procedures, and both studies found that the risk increases as the ASA-PS class increases. In the present study, 71 (84.5%) of the 84 patients that developed hypoxemia were ASA-PS Classes I and II, indicating that the majority of cases did not have serious co-morbid illnesses. The numbers of cases with individual co-morbid illnesses that could affect the cardiorespiratory dynamics or drug metabolism of the sedatives were as follows: 11 respiratory disease patients; five cardiovascular disease patients; five kidney disease patients; and 24 liver disease patients. It must also be emphasized that there were 48 patients (57.1%) that did not have any of the above mentioned illnesses (Table 3). A BMI < 18.5 kgm^−2^ is also considered to be a risk factor^18^; however, 74 of the 84 examined patients had a mean BMI of 25.6 (14.1 to 35.9) kgm^−2^, and there were only three patients with a BMI < 18.5 kgm^−2^. Like emaciation, obesity is thought to pose a major perioperative airway challenge in association with obstructive sleep apnea syndrome. But only 11 patients have a BMI > 30 kgm^−2^. According to reports to date, ASA-PS Classes III and IV and BMI < 18.5 kgm^−2^ were considered risk factors related to the onset of hypoxemia. However, they are not definitive factors that can predict the onset of hypoxemia. Therefore, stratifying the patients according to these factors as a preventive measure for hypoxemia does not ensure safety. These factors should be regarded more as useful factors in determining the adequacy of sedative use. ECOG-PS assesses performance status, and 73 (86.9%) of 84 patients were categorized as Grades 0 and 1. The performance status of the cases that developed hypoxemia was by no means poor. There is a need to fully recognize that the majority of the cases that developed hypoxemia had good performance status and no risk factors, such as co-morbid illnesses, ASA-PS Class III/IV, or low body weight. Furthermore, when the scope of the examination of the 84 cases that showed onset of hypoxemia was expanded to include EGD records at our institution up to July 31, 2017, the records showed that, of the 41 patients that had been administered the same type and dose of sedative, 29 did not develop hypoxemia (Table 1). Once again, this fact highlights the difficulty in predicting the onset of hypoxemia from patient background characteristics. The one case that developed hypoxemia after a long postprocedural gap (60 minutes after the end of the EGD procedure) was categorized as ASA-PS Class I without any co-morbid illness, and the BMI was 27.8 kgm^−2^. There was also a record of a case that was given the same type and dose of sedative without developing hypoxemia. When the 84 cases that developed hypoxemia were divided into three groups depending on the onset of hypoxemia (intraprocedural, at the end of the procedure, and during recovery), and one-way analysis of variance was performed to determine whether there were significant differences in age, BMI, ASA-PS and ECOG-PS, the data did not show differences among the parameters (P = 0.098, 0.098, 0.350, and 0.083, respectively). In other words, it is difficult to predict the timing of onset of hypoxemia based on these patient background parameters.

Several limitations of the present study must be acknowledged. First, this was a retrospective, cross-sectional study. Second, since cases were included in the analysis from our single institution, selection bias may have affected results especially in regards to patient characteristics and sedative choice. Third, since records of EGD procedures under sedation were retrospectively extracted from the electronic medical records, undescribed data of height and body weight in some cases was an inevitable limitation.

In conclusion, more than half of the hypoxemia cases during EGD procedures with planned moderate sedation occurred during the recovery period, and the frequency of occurrence (1.1%) was high. Lack of risk factors such as serious co-morbid illness, low body mass index, and history of sedative-related hypoxemia is no guarantee that hypoxemia will not occur. Therefore, continuous monitoring of oxygen saturation by pulse oximetry with an audible alarm is more important during the recovery period and is recommended in all procedures.

## Methods

This retrospective study was approved by the institutional review board of Saiseikai Kanazawa Hospital (H28-25) and conducted in accordance with the ethical standards described in the latest revision of the Declaration of Helsinki. Informed consent for participation was obtained in the form of an opt-out in-hospital notice.

For endoscopic procedures under planned moderate sedation, our institution has long been conducting intraprocedural monitoring using pulse oximetry with an audible alarm to monitor SpO_2_ and pulse rate. However, as of April 1, 2015, in addition to intraprocedural monitoring, SpO_2_ and pulse rate are continuously monitored through a postprocedural central monitoring system even after the patients are transferred to recovery beds. This system ensures that immediate action can be taken if patients develop hypoxemia, bradycardia, or tachycardia. When hypoxemia is seen, oxygen is immediately administered upon the instructions of the attending physician or endoscopist.

All outpatient cases that underwent EGD procedures with sedation at our institution between April 1, 2015 and December 31, 2016 were included in the analysis. Hospitalized patients were excluded because their postprocedural follow-up took place when they returned to their hospital beds. Records of EGD procedures under sedation were retrospectively extracted from the electronic medical records and were evaluated based on age, sex, and sedatives and analgesics used. From these records, the cases that developed hypoxemia were extracted. Patients who had been receiving oxygen due to conditions such as respiratory failure before the procedures were excluded. As patient background characteristics, age, sex, height, body weight, ECOG-PS, and co-morbid illnesses (respiratory, cardiovascular, kidney, and liver diseases) were extracted. All co-morbid illnesses were assessed based on the ASA-PS classification system. The following parameters were also examined: type and dose of sedatives and analgesics used; the starting and ending times of EGD procedures; starting and ending times of oxygen administration; dose and method of oxygen administration; end time of recovery care; treatments other than oxygen supplementation, such as antagonist agents like flumazenil and tracheal intubation; and outcomes (remission, shift in treatment such as hospitalization, death, etc.). As for hypoxemia cases, EGD procedures conducted between January 1, 2017 and July 31, 2017 were also examined.

Moderate sedation as defined by the ASA^4^ was planned for EGD, and actual practices were carried out in compliance with the guidelines issued by the JSA^19^. According to these guidelines, midazolam should ideally be administered under the supervision of an anesthesiologist. As it mandates an intravenous (IV) line, midazolam is not considered a first-line agent at our institution. Therefore, in principle, diazepam is used for EGD procedures with planned moderate sedation without an IV line, and the standard doses for men and women are 7.5 mg and 6.5 mg, respectively. These standard doses correspond with the initial dose of 5–10 mg indicated in the Multisociety Sedation Curriculum for Gastrointestinal Endoscopy (MSCGE) in 2012^20^. It is intravenously administered by a nurse under the supervision of an endoscopist who is not an anesthesiologist. No sedatives are required or dose reduction is used in elderly patients, extremely emaciated patients, patients with liver, kidney, or respiratory failure, and in hemodynamically unstable patients. In patients who experienced extreme pain in previous procedures, the dose of diazepam is increased or midazolam is used, and in addition to sedatives, pentazocine, which is an analgesic, may be administered. When midazolam is used, an IV line needs to be secured, and the initial diluted dose is slowly given intravenously. Additional doses are administered until moderate sedation is achieved. When using sedatives, arrangements to secure the airway of patients in preparation for potential respiratory arrest are always in place, and flumazenil is readily available.

EGD procedures are performed by two people, the endoscopist and an assisting nurse. We do not follow the guidelines established by the ESGE in 2008^2^, which state that one person in addition to the endoscopist and the assisting nurse, who is not involved in the intervention, must be present. However, all patients are monitored for oxygen saturation and pulse rate using pulse oximetry with an audible alarm during and at the end of procedure [MUE200 (Olympus Medical Systems, Tokyo, Japan), Vismo (Nihon Kohden, Tokyo, Japan), or Moneo BP-88 (COLIN, Tokyo, Japan)]. When the endoscopic procedure is completed, patients are transferred to a recovery bed placed in a curtained-off area inside the endoscopy room for monitoring. Continuous central-monitoring during the recovery period is conducted with five Nellcor N-BSJP monitors and a SAT-MeSSAGE wireless module (Covidien Japan, Tokyo, Japan). The audible alarms are set to be triggered intraprocedurally and postprocedurally including during the recovery period when SpO_2_ drops below 90% or when pulse rates rise above 100 bpm or fall below 50 bpm. There are no dedicated healthcare personnel to monitor the recovering patients. While patients who develop hypoxemia and high-risk patients are appropriately observed by the healthcare personnel in the endoscopy room, there are no standard periodic rounds or monitoring conducted at regular intervals for all cases. When an alarm indicating SpO_2_ < 90 or pulse irregularities sounds, a nurse immediately assesses the condition of the patient. When respiratory depression due to oversedation is observed, the nurse prompts the patient to wake up through stimuli such as calling his name and shaking his body and to place the patient in a lateral position to relieve airway obstruction by glossoptosis. In addition, if SpO_2_ does not increase or decreases soon again, the nurse responds by administering oxygen as hypoxemia after informing the endoscopist or the attending physician and reinforces monitoring. SpO_2_ reduction due to sensor disengagement or temporary coughing is excluded by this observation.

Cases that developed hypoxemia were divided into three groups depending on the onset of hypoxemia (intraprocedural, at the end of the procedure, and during recovery), and one-way analysis of variance was performed to determine the predictive factors associated with the timing of the onset of hypoxemia.

## Data Availability

The datasets generated and/or analyzed during the current study are not publicly available due to including personal information, but are partially available from the corresponding author on reasonable request.
